# Long non‐coding RNA CASC15 enhances learning and memory in mice by promoting synaptic plasticity in hippocampal neurons

**DOI:** 10.1002/EXP.20230154

**Published:** 2024-03-28

**Authors:** Yuankang Zou, Bo Gao, Jiaqiao Lu, Keying Zhang, Maodeng Zhai, Ziyan Yuan, Michael Aschner, Jingyuan Chen, Wenjing Luo, Lei Wang, Jianbin Zhang

**Affiliations:** ^1^ Department of Occupational and Environmental Health and the Ministry of Education Key Lab of Hazard Assessment and Control in Special Operational Environment, School of Public Health Fourth Military Medical University Xi'an China; ^2^ Institute of Orthopaedic Surgery Xijing Hospital Fourth Military Medical University Xi'an China; ^3^ Department of Urology Xijing Hospital Fourth Military Medical University Xi'an China; ^4^ Institute of Medical Information and Library Chinese Academy of Medical Sciences and Peking Union Medical College Beijing China; ^5^ Department of Molecular Pharmacology Albert Einstein College of Medicine Bronx New York USA; ^6^ Department of Medical Research Center, Clinical Medical College Yangzhou University Yangzhou China

**Keywords:** Alzheimer's disease, CASC15, FMR1, long noncoding RNA, NTF3, synaptic plasticity

## Abstract

Alzheimer's disease (AD) is a debilitating systemic disorder that has a detrimental impact on the overall well‐being of individuals. Emerging research suggests that long non‐coding RNAs play a role in neural development and function. Nevertheless, the precise relationship between lncRNAs and Alzheimer's disease remains uncertain. The authors' recent discoveries have uncovered an unconventional mechanism involving the regulation of synaptic plasticity and the functioning of the hippocampal fragile X mental retardation protein 1 (FMR1)—neurotrophin 3 (NTF3) pathway, which is mediated by cancer susceptibility candidate 15 (*CASC15*). Subsequently, functional rescue experiments were performed to illustrate the efficient delivery of exosomes harboring a significant amount of *2610307p16Rik* transcripts, which is the murine equivalent of human *CASC15*, to the hippocampal region of mice. This resulted in significant improvements in synaptic morphological plasticity and cognitive function in *APP/PS1* mice. Given the pivotal involvement of *CASC15* in synaptic plasticity and the distinctive regulatory mechanisms of the CASC15‐FMR1‐NTF3 axis, *CASC15* emerges as a promising biomarker for Alzheimer's disease and may even possess potential as a feasible therapeutic target.

## Graphical abstract

There is an increasing body of evidence suggesting the involvement of long non‐coding RNAs (lncRNAs) in neural development and function. Nevertheless, the relationship between lncRNAs and Alzheimer's disease (AD) remains uncertain. Previous genome‐wide association studies (GWAS) have linked cancer susceptibility candidate 15 (*CASC15*) with AD. This study aims to comprehensively investigate the characteristics and mechanisms underlying *CASC15*‐mediated synaptic plasticity in hippocampal neurons through the FMR1‐NTF3 axis.

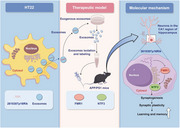

## 1 INTRODUCTION

Neurodegenerative diseases, such as AD, present an unresolved and formidable global issue within the medical field, exerting a significant impact on both social and economic progress.^[^ ^1^ ^]^ It occurs primarily in the elderly (65 years or older) and is characterized by progressive neuronal loss, leading to severe impairment of brain function.^[^ ^2^, ^3^, ^4^, ^5^ ^]^ AD, manifested as learning and memory (L&M) loss and cognitive decline,^[^ ^6^ ^]^ accounts for approximately 50%–75% of all cases of dementia. It is estimated that 44 million people worldwide suffer from AD, with an incidence of 4.6 million new cases each year with a higher prevalence in in the elderly.^[^ ^7^, ^8^ ^]^ AD has a particular specific feature that the risk of suffering doubles every 5 years starting at age 60.^[^ ^8^, ^9^ ^]^ The accumulation of amyloid‐β (Aβ) aggregates and hyper‐phosphorylated tau protein, together contribute to neurofibrillary tangles (NFTs) and synaptic dysfunction, are important hallmarks of AD pathology.^[^ ^8^, ^10^, ^11^, ^12^, ^13^, ^14^, ^15^, ^16^ ^]^ Regrettably, thus far, no targeted interventions have been devised for the management of AD apart from medications aimed at providing temporary alleviation of symptoms. Consequently, the identification and prompt treatment of individuals afflicted with AD have emerged as pivotal factors in clinical intervention. Synaptic plasticity the activity‐dependent change in neuronal connection strength, has long been considered an important component of learning and memory.^[^ ^17^ ^]^ Synaptic plasticity changes in the early stages of AD and then progresses to loss of synaptic function in the later stage of the disease.^[^ ^18^, ^19^, ^20^, ^21^ ^]^ Therefore, further understanding of the molecular mechanisms behind changes in synaptic plasticity in AD pathophysiology and blocking the core pathogenic targets may provide a promising application for early clinical treatment of AD.

Long non‐coding RNAs (LncRNAs), a heterogeneous class of endogenous non‐coding RNAs (ncRNAs) possessing more than 200 nucleotides, are accompanied by a confining ability to encode proteins.^[^ ^22^ ^]^ Compared to protein‐coding potential genes, lncRNAs have unique structures and biochemical characteristics and participate in neural development,^[^ ^23^ ^]^ differentiation,^[^ ^24^ ^]^ synaptic plasticity^[^ ^25^, ^26^ ^]^ and other biological functions through a variety of potential mechanisms throughout the eukaryotic genomes.^[^ ^27^, ^28^ ^]^ It has been reported that about 40% of lncRNAs are specifically expressed in the brain, but only a small portion of abnormally lncRNAs have been well characterized as being associated with many neurodegenerative diseases.^[^ ^29^, ^30^, ^31^, ^32^, ^33^ ^]^ A large number of abnormal lncRNAs were found to be expressed in the hippocampal region of *APP/PS1* transgenic mice,^[^ ^34^ ^]^ and the same phenomenon was found in the transcriptome analysis of human brain.^[^ ^35^, ^36^ ^]^ lncRNA cancer susceptibility candidate 15 (*CASC15*) is highly enriched in the hippocampus, plays an important role in cognitive function, and its expression is usually declined in the brain of AD patients.^[^ ^37^, ^38^ ^]^ We find that the disruption of *2610307p16Rik*, a murine counterpart of human *CASC15*, leads to aberrant synaptic plasticity and compromised neuronal function in mice. Recent findings made a strong case for the regulation of protein synthesis by presynaptic Fragile X mental retardation protein (FMRP) in presynaptic structural and functional plasticity at excitatory presynapses.^[^ ^39^ ^]^ Additional research groups have discovered that the expression of neurotrophin mRNA in hippocampal neurons of *FMR1*‐knockout mice is elevated and predominantly localized within the dendrites of neurons. These findings suggest that the presence of FMR1 protein in hippocampal neurons can impede the transportation of neurotrophin mRNA, resulting in a down‐regulation of its expression.^[^ ^40^ ^]^ The neurotrophin family exerts a significant influence on the facilitation of learning and memory through the regulation of synaptic plasticity and long‐term potentiation (LTP) in hippocampal neurons. Among the neurotrophin family, neurotrophin 3 (NTF3) holds considerable importance.^[^ ^41^, ^42^ ^]^ The results of our study suggest that the preservation of synaptic plasticity and the maintenance of learning and memory capabilities are dependent on the substantial participation of *CASC15*. This involvement effectively inhibits the expression of FMRP, thereby facilitating the elevation of NTF3 levels.

Exosomes are extracellular vesicles native to the endosomal pathway which convey endogenous bioactive molecules, such as mRNA, ncRNA, and proteins, etc., from their parental cells.^[^ ^43^, ^44^, ^45^, ^46^ ^]^ They carry specific cargo, which are involved in cellular communication, in almost all types of brain cells, implicating physiological and pathological associations of the brain.^[^ ^47^, ^48^, ^49^ ^]^ In addition to propagating within cells, exosome‐mediated signals travel widely throughout the brain via the cerebrospinal fluid.^[^ ^50^, ^51^, ^52^ ^]^ It has also been demonstrated that exosome contents can delay dementia through restoring neuronal function or clearing Aβ.^[^ ^48^, ^53^ ^]^ Further, we find that *2610307p16Rik* is not only expressed in neurons, but also specifically enriched in exosomes secreted by mouse hippocampal neuronal HT22 cells. Additionally, we discover that synaptic morphological plasticity and cognitive ability of *APP/PS1* mice can be enhanced by delivering exosomes enriched in *2610307p16Rik* transcripts to mouse cerebral cortex and hippocampus. Taken together, *CASC15* has a wide range of applications as a biomarker for AD, even as a potential therapeutic target through the use of exosome delivery. The phenomenon of synaptic plasticity undergoes alterations during the initial phases of AD, subsequently leading to a decline in synaptic function during the later stages of the ailment. Genome‐wide association studies have established a correlation between AD and the presence of *CASC15*. The aim of this study was to investigate the role and mechanism of *CASC15* in the pathogenesis of AD and evaluate *CASC15*, along with the mouse homologous gene *2610307p16Rik*, as a potential therapeutic approach for AD by preserving synaptic plasticity.

## 2 RESULTS AND DISCUSSION

### 2.1 *CASC15* is highly expressed in hippocampal tissue

**FIGURE 1:**
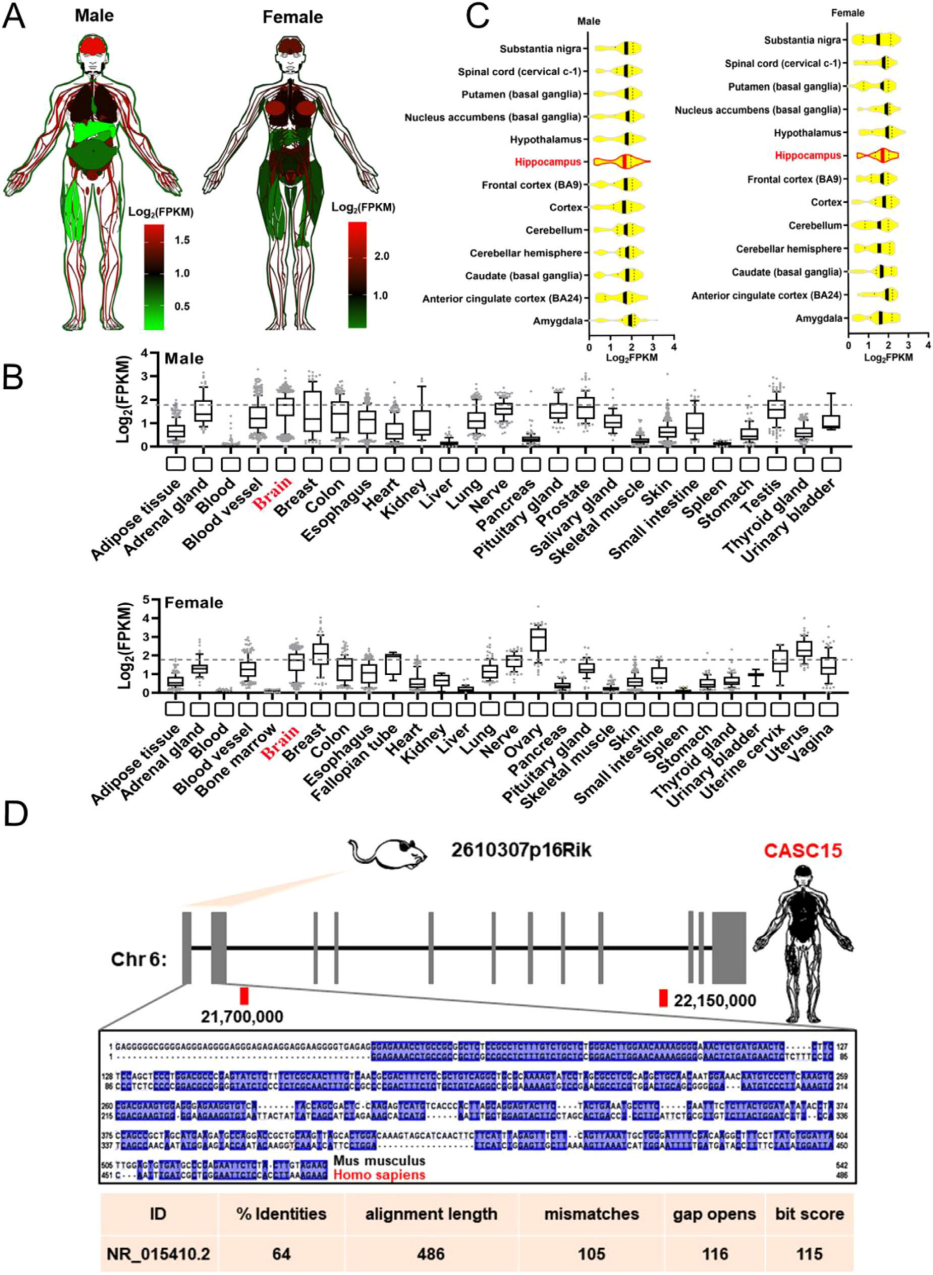
*CASC15* is highly expressed in hippocampal tissue. (A) Human body plot illustrating the expression of *CASC15* in human tissues from the genotype‐tissue expression (GTEx) project; values shown are the Log2^FPKM^ values by tissue. (B) Barplots showing expression of the *CASC15* in human tissues from the GTEx project; brain expression is outlined in red. (C) Box plots showing expression of the *CASC15* gene in human brain subregions from the GTEx project; hippocampus expression is outlined in red. (D) Schematic representation of alignment signatures found for mouse and the orthologous human *CASC15* using slncky Evolution Browser; the conserved region between mouse and human is emphasized and the conserved sequences between mammals are underlined in blue. The conservation values of BLAST were summarized in the bottom table.

To investigate the potential functions of *CASC15* in neural development under normal physiological conditions, the expression of *CASC15* in normal human tissues and organs were analyzed using GTEx database. It was found that *CASC15* is widely expressed in many tissues (Figure 1A,B) and highly enriched in various human brain regions, including hippocampal tissues (Figure 1C). To track the homologous of those lncRNA from mice, the evolutionary conservation tests were conducted. Results suggested that *2610307p16Rik* is the homologous molecule of *CASC15* in mice, and both molecules share a highly evolutionary conserved 5′ ends as well (Figure 1D). This indicated that *2610307p16Rik* may also bear a critical role in paralleled with *CASC15* in sustaining the function of hippocampal tissue.

### 2.2 *2610307p16Rik* knockout mice have shown impairment in learning and memory functions

**FIGURE 2:**
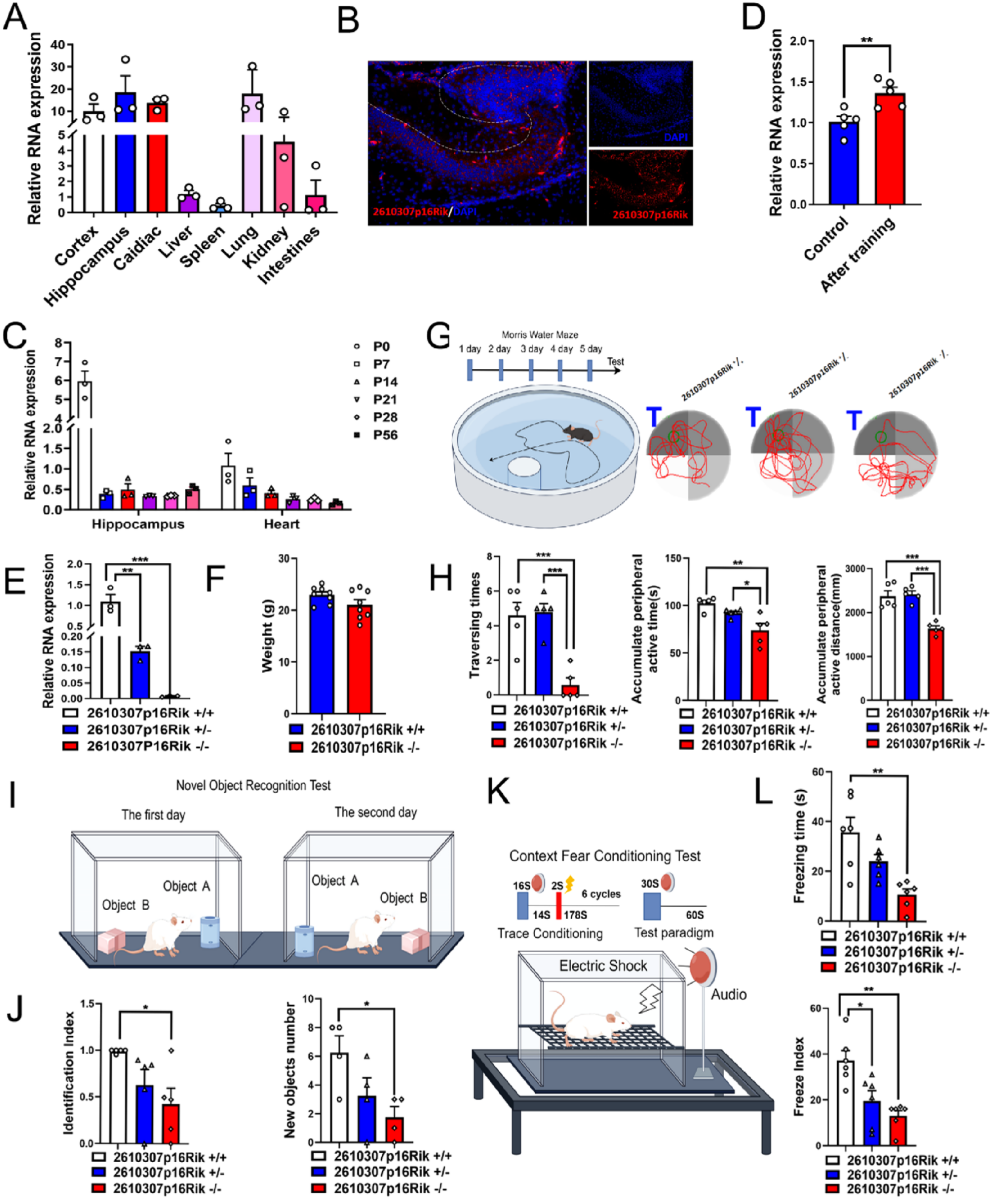
*2610307p16Rik* knockout mice have shown impairment in learning and memory functions. (A) Level of enrichment of *2610307p16Rik* in various tissues and organs of mice was assessed by qRT‐PCR (*U1snRNA* was used as control), and data were normalized to the expression of liver (mean ± SEM, *n* = 3). (B) The expression of *2610307p16Rik* in mouse hippocampus was detected by RNA‐FISH, scale bar: 10 µm. (C) The expression of *2610307p16Rik* in the hippocampus and heart at different stages of nervous system development was measured by qRT‐PCR (*U1snRNA* was used as control), and data were normalized to the expression of P 0 heart (mean ± SEM, *n* = 3). (D) Relative expression levels of *2610307p16Rik* before and after water maze training, and data were normalized to the expression of control group before water maze training (mean ± SEM, *n* = 5). Student′s *t*‐test **p <  0.01. (E) Relative expression levels of *2610307p16Rik* in hippocampal tissues of *2610307p16Rik* wild‐type, heterozygous, and knock‐out mice, and data were normalized to the expression of wild‐type group (mean ± SEM, *n* = 3). Student′s *t*‐test **p <  0.01, ***p <  0.001. (F) Body weight of *2610307p16Rik* wild‐type and knock‐out mice (mean ± SEM, *n* = 8). (G) Model diagram of water maze and schematic diagram of space exploration experiment. (H) Number of platform crossing times, accumulate peripheral active time and distance in space exploration experiment of Morris water maze (mean ± SEM, *n* = 5). Student′s *t*‐test *p <  0.05, **p <  0.01, ***p <  0.001. (I) Model diagram of novel object recognition task. (J) Discriminate index, and exploring numbers on new objects of *2610307p16Rik* wild‐type, heterozygous, and knock‐out mice in novel object recognition task (mean ± SEM, *n* = 5). Student′s *t*‐test *p <  0.05. (K) Model diagram of context fear conditioning test. (L) Freeze index, and freezing time of *2610307p16Rik* wild‐type, heterozygous, and knock‐out mice in context fear conditioning test (mean ± SEM, *n* = 6). Student′s *t*‐test *p <  0.05, **p <  0.01.

To test if the tissue‐specific expression of *2610307p16Rik* is consistent with that of *CASC15*, the expression level of *2610307p16Rik* in various tissues and organs of adult C57BL/6 mice was examined using qPCR. Results showed that *2610307p16Rik* was highly expressed in brain tissue compared with the expression of liver (Figure 2A), which was similar to the human expression profiles. Further, ISH and FISH tests were conducted to confirm that *2610307p16Rik* is highly enriched in the CA1 region neurons of the hippocampus originated from C57 mice (Figure 2B and Figure S1A). Intriguingly, it was found that the expression level of *2610307p16Rik* was dynamic during the neurodevelopmental process of mice (Figure 2C). Compared with P0 hearts, *2610307p16Rik* was expressed at higher levels in the hippocampus and cerebral cortex (Figure 2C and Figure S1B).

In assuming that the high enrichment of *2610307p16Rik* in the mouse hippocampus is associated with mice L&M, C57BL/6 mice were trained with Morris water maze for a period of time and the expression level of *2610307p16Rik* in the hippocampus was up‐regulated to a certain degree (Figure 2D). To investigate the specific role of *2610307p16Rik* in L&M process, *2610307p16Rik* knockout mouse model was constructed using CRISPR‐Cas9 and, as a result of qPCR assay, it was noted that *2610307p16Rik* expression levels in heterozygous mice were in between wild type and knockout mice (Figure 2E). Meanwhile, there were no significant phenotypic differences in limb development, body weight, visual acuity, hearing, and brain tissue structure between *2610307p16Rik* knockout mice and wild type group (Figure 2F, Figure S1C,D, and data not shown). However, a series of behavioral experiments revealed that *2610307p16Rik* knockout group's performance in L&M was significantly impaired (Figure 2G–L and Figure S1E,F) compared to the wild type group, underlining the role of *2610307p16Rik* in the L&M process.

### 2.3 Loss of *2610307p16Rik* impairs synaptic plasticity of hippocampal CA1 region in mice

**FIGURE 3:**
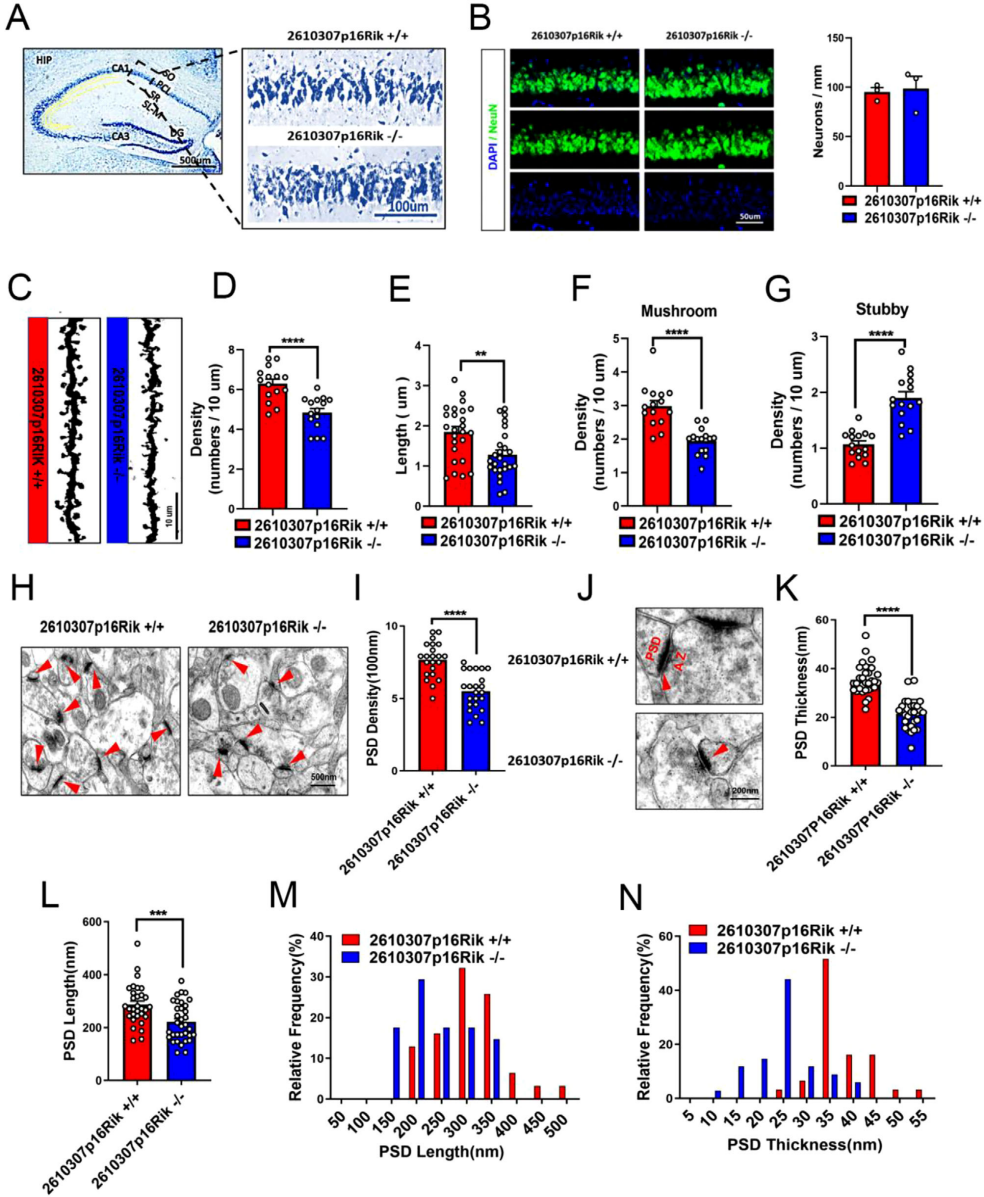
Loss of *2610307p16Rik* impairs synaptic plasticity in the CA1 region of hippocampal in mice. (A) Nissler staining was conducted to test the number of neurons in the CA1 region of *2610307p16Rik* wild‐type and knock‐out mice hippocampus, scale bar: 100 µm. (B) NeuN positive cells in the CA1 region of *2610307p16Rik* wild‐type and knock‐out mice hippocampus was detected by immunohistochemical staining. (C) Golgi staining analyses were used to observe the changes of neuronal dendritic spines in the CA1 region of *2610307p16Rik* wild‐type and knock‐out mice hippocampus, scale bar: 10 µm. (D) Quantitative analysis of the density of neuronal dendritic spines in the CA1 region of hippocampal (mean ± SEM, 5 neurons/3 mice per group). Student′s *t*‐test ****p <  0.0001. (E) Quantitative analysis of the length of neuronal dendritic spines in the CA1 region of hippocampus (mean ± SEM, 5 neurons/3 mice per group). Student′s *t*‐test **p <  0.01. (F) Quantitative analysis of the number of mature Mushroom dendritic spines in the CA1 region of hippocampus (mean ± SEM, 5 neurons/3 mice per group). Student′s *t*‐test ****p <  0.0001. (G) Quantitative analysis of the number of mature Stubby dendritic spines in the CA1 region of hippocampus (mean ± SEM, 5 neurons/3 mice per group). Student′s *t*‐test ****p <  0.0001. (H) Transmission electron microscopy shows synaptic density changes of neurons in the CA1 region of the hippocampus of *2610307p16Rik* knockout mice; the red triangle identifies the synapse, scale bar: 500 nm. (I) Quantitative analysis of the density of synapses in the CA1 region of the hippocampus (mean ± SEM, 7–8 neurons/3 mice per group). Student′s *t*‐test ****p <  0.0001. (J) Transmission electron microscopy was used to illustrate the synaptic thickness changes of neurons in the CA1 region of the hippocampus; the red triangle identifies the synapse, scale bar: 200 nm. (K) Quantitative analysis of the thickness of synapses in the CA1 region of the hippocampus (mean ± SEM, 10–11 neurons/3 mice per group). Student′s *t*‐test ****p <  0.0001. (L) Quantitative analysis of the length of synapses in the CA1 region of hippocampus (mean ± SEM, 10–11 neurons/3 mice per group). Student′s *t*‐test ****p <  0.0001. (M) The relative frequency of the length of synapses in the CA1 region of the hippocampus. (N) The relative frequency of the thickness of synapses in the CA1 region of the hippocampus.

Based on the findings obtained from behavioral experiments, the present study employed Nissler and immunohistochemical staining techniques to examine the potential impact of knockout *2610307p16Rik* on neuronal quantity within the CA1 region of the hippocampus. There were no apparent changes in the number of neurons in the CA1 region of hippocampus after *2610307p16Rik* knockout (Figure 3A,B). Golgi staining analyses were used to further illustrate the knockout effect of gene on the neural plasticity, and it was observed that the number and length of neuronal dendritic spines was significantly decreased, the number of mature Mushroom dendritic spines was significantly decreased, and the number of immature Stubby dendritic spines was significantly increased in the hippocampal CA1 region (Figure 3C–G). Transmission electron microscopy was used to illustrate the synaptic morphological changes of neurons in the CA1 region of *2610307p16Rik* knockout mice. Synaptic density was reduced, postsynaptic membrane length was shorter, and postsynaptic membrane thickness was thinner (Figure 3H–N). Taken together, the results suggested that *2610307p16Rik* is involved in L&M process through exerting effects on neural plasticity in the CA1 region of the mouse hippocampal tissue.

### 2.4 Hippocampal CA1 neurons lost synaptic function behind *2610307P16Rik* knockout

**FIGURE 4:**
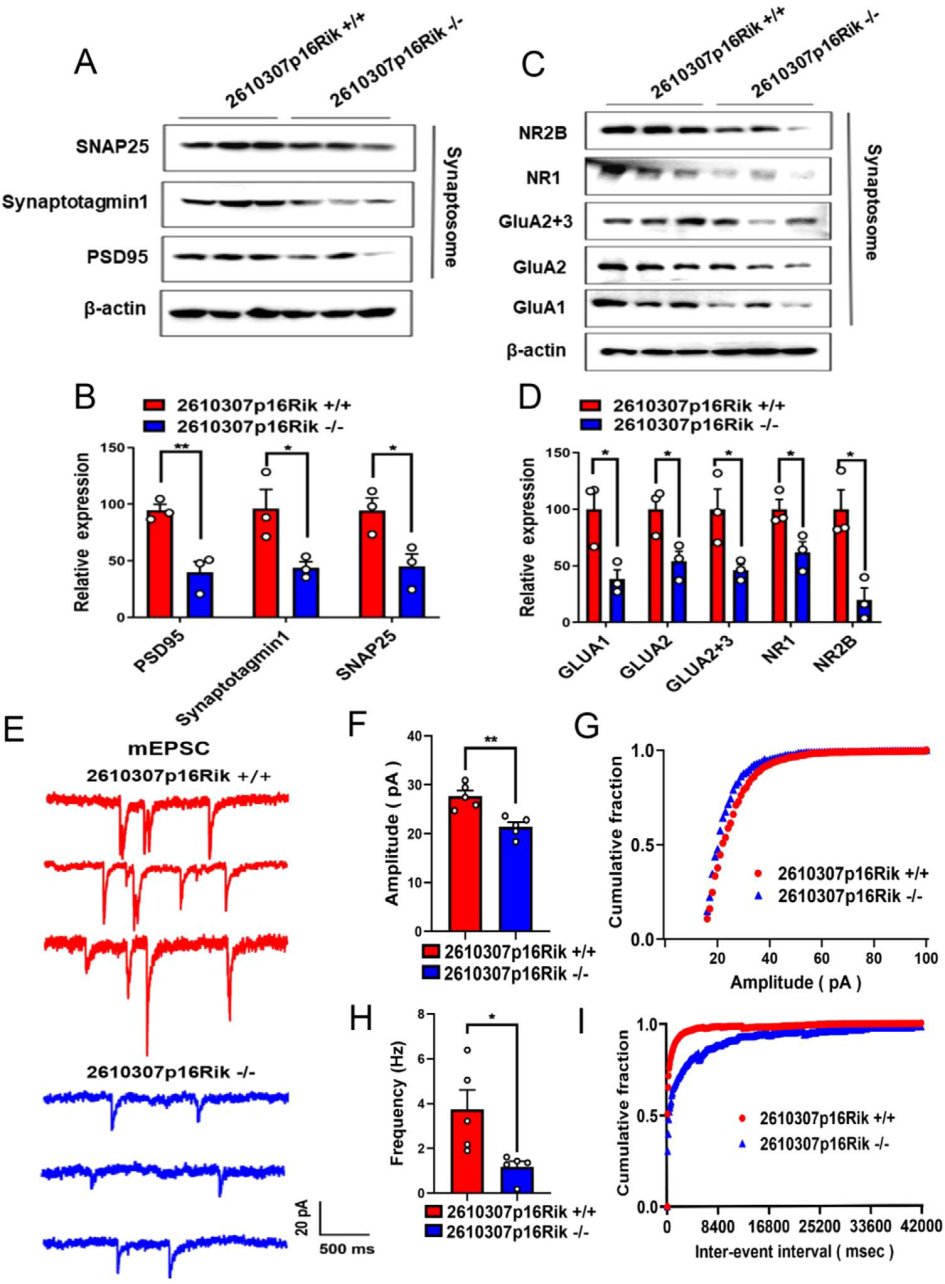
Hippocampal CA1 neurons lost synaptic function behind *2610307P16Rik* knockout. (A) Western blot was conducted to detect the expression levels of PSD95, SNAP25 and Synaptotagmin1. (B) Quantitative analysis of the expression levels of PSD95, SNAP25 and Synaptotagmin1, and data were normalized to the expression of *2610307p16Rik* wild‐type group (mean ± SEM, *n* = 3). Student′s *t*‐test *p <  0.05, **p <  0.01. (C) Western blot was conducted to detect the expression levels of AMPA and NMDA receptor protein. (D) Quantitative analysis of the expression levels of NR2B, NR1, GluA2+3, GluA2, GluA1, and data were normalized to the expression of *2610307p16Rik* wild‐type group (mean ± SEM, *n* = 3). Student′s *t*‐test *p <  0.05, **p <  0.01. (E) Representative traces of mEPSC in the CA1 region of hippocampal. (F,G) Quantification of mEPSC amplitude (mean ± SEM, *n* = 3). Student′s *t*‐test **p <  0.01. (H,I) Quantification of mEPSC frequency (mean ± SEM, *n* = 3). Student′s *t*‐test *p <  0.05.

The normal physiological functions of neurons depend mainly on pre‐ and post‐synaptic membrane receptors and synaptic vesicle release.^[^ ^54^, ^55^, ^56^, ^57^ ^]^ Western blot experiments were conducted to see whether the normal physiological functions were impaired after *2610307p16Rik* knockout. It came out that the expression levels of pre and post synaptic membrane, and synaptic vesicle protein in hippocampal neurons were significantly decreased, indicating synaptic damage in hippocampal neurons after knockdown (Figure 4A,B). This part of the results was consistent with the results of electron microscopy (Figure 3H,J). Numerous studies have shown that AMPA and NMDA receptor proteins are abundant in the pre and post synaptic membrane. As expected, the results also revealed that the expression levels of AMPA and NMDA receptor protein in the pre‐ and post‐synaptic membranes of hippocampal neurons were significantly decreased after *2610307p16Rik* knockout (Figure 4C,D). Patch clamp technique was used to record the mEPSC release of the hippocampal CA1 region after *2610307p16Rik* knockout, and it turned out that both the amplitude and the frequency of mEPSC were significantly decreased after operation, showing that neuronal functions were impaired (Figure 4E–I). These results suggested that knock out *2610307p16Rik* affects the synaptic plasticity in the CA1 region of mice tissues, leading to functional damage of neurons.

### 2.5 NTF3 and FMR1 are key regulatory factors downstream of *2610307p16Rik*

**FIGURE 5:**
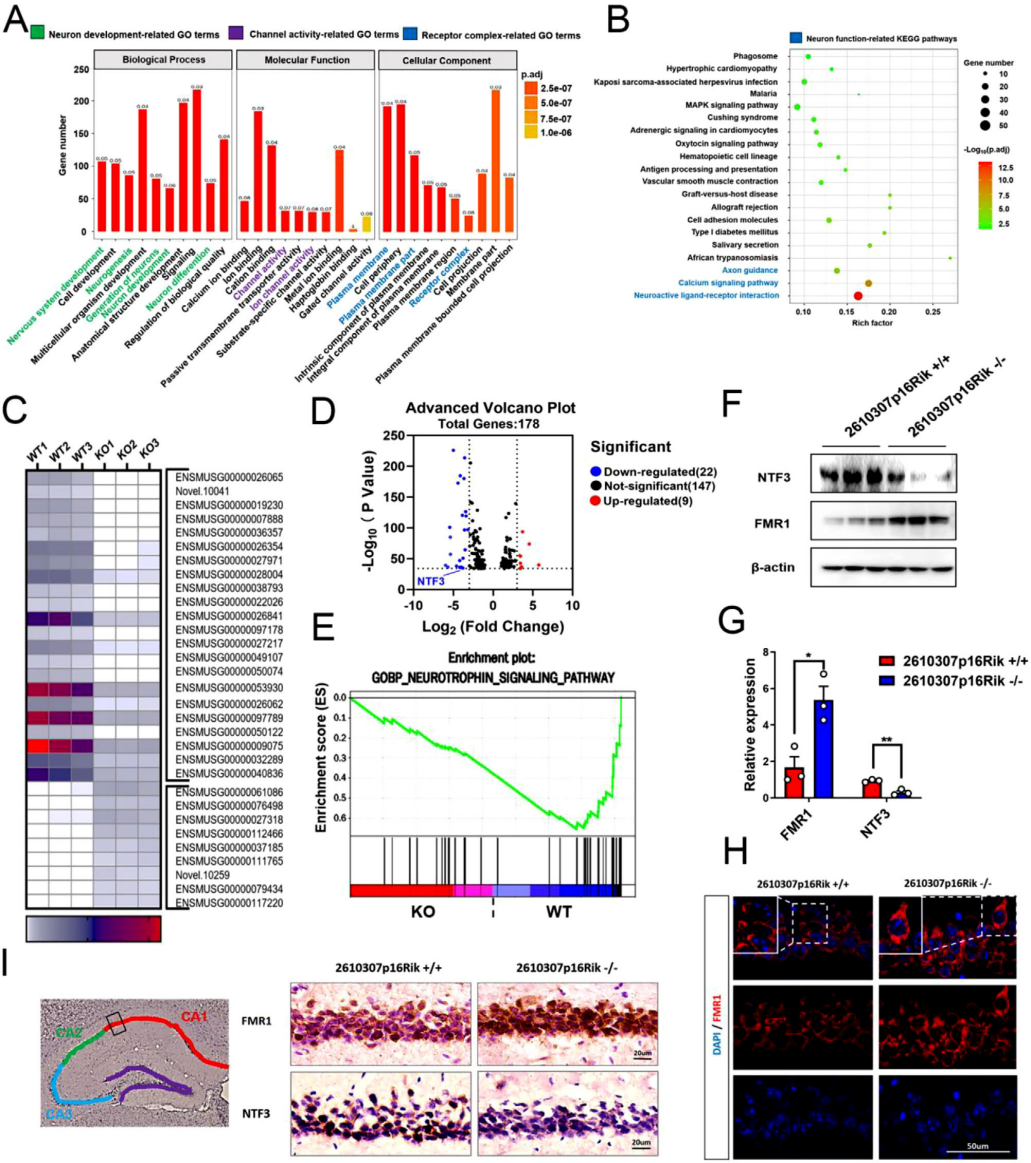
NTF3 and FMR1 are key regulatory factors downstream of *2610307p16Rik*. (A) GO analysis of RNA‐seq data on altered expression of genes. (B) KEGG analysis showed the top 20 signaling pathways of RNA‐seq data on altered expression of genes. (C) Heat map analysis showed the altered expression of genes of RNA‐seq data, |log_2_^(Fold Change)^ | > 3; log_10_^(*p* value)^ > 34. (D) Volcano plots of RNA‐seq data, |log_2_^(Fold Change)^ | > 3; log_10_^(*p* value)^ > 34. (E) GSEA enrichment analysis of RNA‐seq data showed the neurotrophin signaling pathway. (F) Western blot was conducted to detect the expression levels of NTF3 and FMR1 of the hippocampal specimens from WT and *2610307p16Rik* KO transgenic mice. (G) Quantitative analysis of the expression levels of NTF3 and FMR1, and data were normalized to the expression of *2610307p16Rik* wild‐type group (mean ± SEM, *n* = 3). Student′s *t*‐test *p <  0.05, **p <  0.01. (H) Immunostaining for FMR1 in the CA1 region of the hippocampus. (I) Immunohistochemistry was performed to detect the expression levels of NTF3 and FMR1 in the CA1 region of the hippocampus.

To elucidate the specific molecular mechanism by which *2610307p16Rik* affects the synaptic plasticity of neurons in the CA1 region of mouse hippocampus, hippocampal tissue specimens from wild type and *2610307p16Rik* knockout mice were collected for RNA‐seq assay, GO and KEGG passage enrichment analysis. The results revealed that the *2610307p16Rik* knockout group was significantly enriched in neuronal development and related pathways compared with the *2610307p16Rik* wild type group (Figure 5A,B). Further, GSEA enrichment analysis indicated that the neurotrophin signaling pathway was generally down‐regulated and RNA‐seq assays captured significant down‐regulated expression of NTF3, a neurotrophic factor (Figure 5C–E). Meanwhile, to confirm the previous RNA‐seq assay results, Western blot and IHC experiments were performed, which showed in depth that the expression of RNA binding protein FMR1 was significantly up‐regulated and NTF3 was down‐regulated after *2610307p16Rik* knockout (Figure 5F–I).

### 2.6 CASC15‐FMR1 axis is involved in regulating the expression of neurotrophic factor NTF3

**FIGURE 6:**
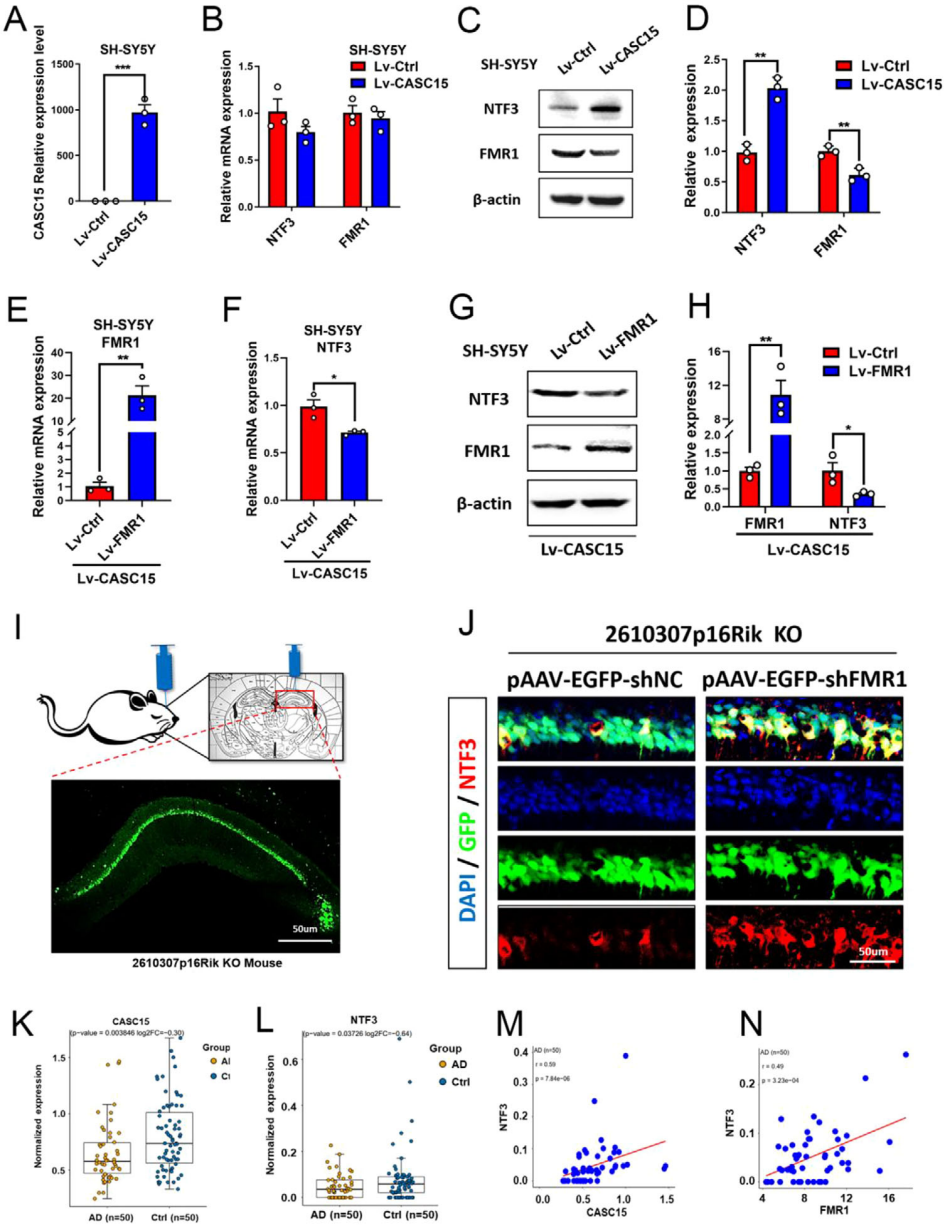
CASC15‐FMR1 axis is involved in regulating the expression of neurotrophic factor NTF3. (A) qRT‐PCR was used to detect the expression of *CASC15* in human‐derived neuroblastoma cell line SH‐SY5Y, and data were normalized to the expression of Lv‐Ctrl group (mean ± SEM, *n* = 3). Student′s *t*‐test ***p <  0.001. (B) qRT‐PCR was used to detect the expression levels of *NTF3* and *FMR1* after *CASC15* overexpression in SH‐SY5Y cells, and data were normalized to the expression of Lv‐Ctrl group (mean ± SEM, *n* = 3). (C) Western blot was conducted to detect the expression levels of NTF3 and FMR1 after *CASC15* overexpression in SH‐SY5Y cells. (D) Quantitative analysis of the expression levels of NTF3 and FMR1, and data were normalized to the expression of Lv‐Ctrl group (mean ± SEM, *n* = 3). Student′s *t*‐test **p <  0.01. (E) qRT‐PCR was used to detect the expression levels of *FMR1* after *FMR1* and *CASC15* overexpression in SH‐SY5Y cells, and data were normalized to the expression of Lv‐Ctrl group (mean ± SEM, *n* = 3). Student′s *t*‐test **p <  0.01. (F) qRT‐PCR was used to detect the expression levels of *NTF3* after *FMR1* and *CASC15* overexpression in SH‐SY5Y cells, and data were normalized to the expression of Lv‐Ctrl group (mean ± SEM, *n* = 3). Student′s *t*‐test **p <  0.05. (G) Western blot was conducted to detect the expression levels of NTF3 and FMR1 after FMR1 and *CASC15* overexpression in SH‐SY5Y cells. (H) Quantitative analysis of the expression levels of NTF3 and FMR1, and data were normalized to the expression of Lv‐Ctrl group (mean ± SEM, *n* = 3). Student′s *t*‐test *p <  0.05, **p <  0.01. (I) Stereotaxic injection pattern and GFP expression in the hippocampus of mice, scale bar: 50 µm. (J) Co‐staining for GFP with NTF3 in the CA1 region of hippocampal after knockdown *FMR1* expression by using pAAV‐EGFP‐*shFMR1* virus in *2610307p16Rik* knock out mice, scale bar: 50 µm. (K,L) Relative RNA levels of *CASC15* and *NTF3* in the 50 clinical AD cases and matched pairs. (M,N) Pairwise correlations among *CASC15*, *NTF3*, and *FMR1* in the 50 clinical AD cases and matched pairs.

Numerous studies have shown that FMR1 is actively involved in the regulation of neuron development^[^ ^58^, ^59^ ^]^ and neuronal synaptic plasticity^[^ ^60^ ^]^ by regulating the expression of another mRNA neurotrophic factor, brain derived neurotrophic factor (*BDNF*), which in turn is involved in the regulation of neuronal development and neuronal synaptic plasticity.^[^ ^61^, ^62^ ^]^ However, as the additional member of the neurotrophin family, the role of NTF3 involved in the regulation of neuronal synaptic plasticity remains to be illustrated. Human‐derived neuroblastoma cell line SH‐SY5Y was acquired to construct *CASC15* over‐expressed cell line. The western blot experiment showed that the expression of FMR1 was significantly down‐regulated, whereas NTF3 was significantly up‐regulated (Figure 6A,C,D). However, mRNA expression levels of *FMR1* and *NTF3* did not change (Figure 6B). Further, cell line that overexpressing both *FMR1* and *CASC15* was constructed to see the effect on NTF3 transcription and protein expression. The results showed that both were decreased in response to *FMR1* and *CASC15* over‐expression (Figure 6E–H). To further verify the regulatory pathway of CASC15‐FMR1 axis on NTF3 in vivo, we examined the expression of NTF3 molecules in the hippocampal CA1 region of *2610307p16Rik* knockout mice by injecting pAAV‐EGFP‐*FMR1* knockdown virus into the hippocampal region. The data revealed that 21 days after injection, the expression of NTF3 was significantly increased in the knockdown group compared to that of the normal group (Figure 6I,J).

### 2.7 *CASC15* has potential value in treating and improving L&M capacity in AD patients

Preliminary studies have demonstrated *CASC15* is involved in hippocampus‐related L&M process by regulating neuronal synaptic plasticity. This intriguingly triggers us to seek for a link between abnormal expression of *CASC15* and AD. Initially, hcluster hierarchical clustering analysis based on a sample of 50 clinical AD cases revealed decreased expression of *CASC15* and *NTF3*, which were closely associated with the development AD (Figure 6K,L). In addition, there was a significantly positive correlation between the expression of *CASC15* and *NTF3* in clinical AD cases (Figure 6M). Although, the expression of *FMR1* did not seem to be associated with development of AD (Figure S2A), there was a negative correlation between the expression of *FMR1* and the expression of both *CASC15* (Figure S2B) and *NTF3* (Figure 6N) molecules.

**FIGURE 7:**
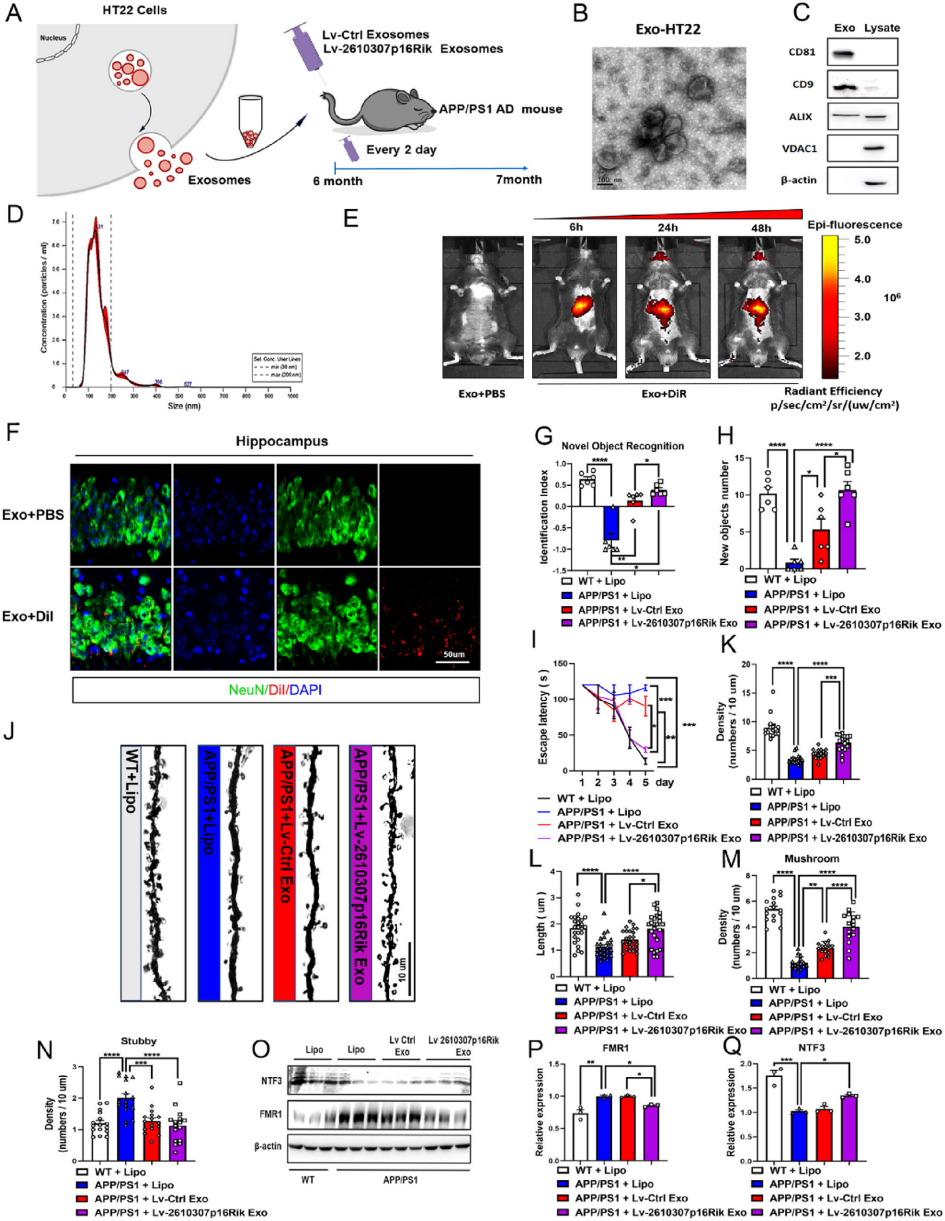
*CASC15* has potential value in treating and improving L&M capacity in AD patients. (A) Model diagram of *APP/PS1* mice treated with exosomes enriched with *2610307p16Rik* transcripts. (B) Exosomes isolated from HT22 cells were detected by transmission electron microscopy, scale bar: 100 nm. (C) Total proteins of exosomes and HT22 cell lysates were prepared, and the levels of CD81, CD9, ALIX and VDAC1 were determined by Western blot. (D) The size of exosomes was assessed by nanoparticle tracking analysis. (E) Fluorescence imaging in the mice showed the distribution of DiR‐labeled exosomes at 6 h/24 h/48 h after injection into the body through the tail vein. (F) Co‐staining for DiI with NeuN in the CA1 region of the hippocampus showed the distribution of DiI labeled exosomes at 48 h after injection into the body through the tail vein, scale bar: 50 µm. (G,H) Discriminate index and exploring numbers on new objects of *APP/PS1* and control mice with different treatment methods in novel object recognition task (mean ± SEM, *n* = 6). Two‐way ANOVA *p <  0.05, **p <  0.01, ****p <  0.0001. (I) Escape latency of *APP/PS1* and control mice with different treatment methods in hidden platform trial of Morris water maze (mean ± SEM, *n* = 6). Two‐way ANOVA **p <  0.01, ***p <  0.001. (J) Golgi staining analyses were used to observe the changes of neuronal dendritic spines in the CA1 region of the hippocampus of *APP/PS1* and control mice with different treatment methods, scale bar: 10 µm. (K) Quantitative analysis of the density of neuronal dendritic spines of in the CA1 region of hippocampal of *APP/PS1* and control mice with different treatment methods (mean ± SEM, 5 neurons/3 mice per group). Two‐way ANOVA ***p <  0.001, ****p <  0.0001. (L) Quantitative analysis of the length of neuronal dendritic spines in the CA1 region of hippocampal of *APP/PS1* and control mice with different treatment methods (mean ± SEM, 5 neurons/3 mice per group). Two‐way ANOVA *p <  0.05, ****p <  0.0001. (M) Quantitative analysis of the number of mature Mushroom dendritic spines in the CA1 region of the hippocampus of *APP/PS1* and control mice with different treatment methods (mean ± SEM, 5 neurons/3 mice per group). Two‐way ANOVA **p <  0.01, ****p <  0.0001. (N) Quantitative analysis of the number of mature Stubby dendritic spines in the CA1 region of hippocampal of *APP/PS1* and control mice with different treatment methods (mean ± SEM, 5 neurons/3 mice per group). Two‐way ANOVA ****p <  0.0001. (O) Western blot was conducted to detect the expression levels of NTF3 and FMR1 of the hippocampal specimens from *APP/PS1* and control mice with different treatment methods. (P,Q) Quantitative analysis of the expression levels of NTF3 and FMR1, and data were normalized to the expression of *APP/PS1*+Lipo group (mean ± SEM, *n* = 3). Two‐way ANOVA *p <  0.05, **p <  0.01, ***p <  0.001.

Advanced studies have revealed that *CASC15* is not only expressed in neurons, but also specifically enriched in its secreted exosomes, indicating that exosome‐mediated *CASC15* delivery may have good potential for the treatment of AD patient in early stage. The mouse‐derived neuronal cell line HT22 was used to constructed the *2610307p16Rik* over‐expression cell line, and purified exosomes were extracted from the *2610307p16Rik* over‐expression cell line. These exosomes were introduced into mice and hippocampal tissues were taken after 48 h of treatment. The qPCR assays revealed the over‐expression efficiency of *2610307p16Rik* in *2610307p16Rik* over‐expression cell line, exosomes and hippocampal tissues (Figure S3A–C). To demonstrate, *APP/PS1* AD mouse model was obtained and exosomes enriched with *2610307p16Rik* transcripts was introduced into hippocampal tissue of mice. Behavioral tests were performed after 4 weeks of operation (Figure 7A). It was noted that most of the purified exosome from neuron were 100–200 nm in diameter, which was in consistent with the diameter profile of exosomes in vivo (Figure 7B–D). After co‐incubation with the purified exosomes using far‐infrared dye DiR, the mice were injected with tail vein. It was observed that DiR‐labeled exosomes could enter the brain tissues of mice 24 h after introduction, and exosomes were still seen to be highly enriched in brain tissue 48 h after initial introduction (Figure 7E). Subsequently, dye DiI was employed and incubated with purified exosomes to investigate the locomotion of exosomes within the CA1 region of the hippocampus in mice. It was found that exosomes were highly enriched in the CA1 region of mouse hippocampus and cortical tissues 48 h after introduction (Figure 7F and Figure 3D), indicating that exosomes can enter hippocampus and remain stable for at least 48 h. Further, exosomes enriched with *2610307p16Rik* transcripts were introduced into mice using the *APP/PS1* AD mouse model and behavioral tests were performed 4 weeks after treatment. Studies showed that new object recognition and spatial learning were significantly improved in *APP/PS1* AD mice treated with exosomes enriched with *2610307p16Rik* transcript compared with their untreated companion (Figure 7G–I and Figure 3E). In brief, these results suggest that exosomes enriched with *2610307p16Rik* transcript can effectively improve L&M ability in AD mice.

Since synapses loss is one of the early onset hallmarks of AD,^[^ ^63^, ^64^ ^]^ improving synaptic plasticity in hippocampal neurons may improve L&M performance in AD mice. To verify this, *APP/PS1* AD mice were treated with exosomes enriched with *2610307p16Rik* transcript, and it was found that these treated mice acquired significantly higher dendritic spine density, longer length, more mature mushroom dendritic spines, and fewer number of immature stubby dendritic spines (Figure 7J–N). The *APP/PS1* AD mouse group treated with *2610307p16Rik* transcript‐rich exosomes showed a significant decrease in FMR1 protein expression and a significant increase in NTF3 protein expression in the hippocampus region (Figure 7O–Q and Figure 3F,G).

## 3 CONCLUSIONS

In this work, the functional deficiency phenotype of *CASC15* was constructed and further studies found that after *2610307p16Rik* knockout, the expression of NTF3, a key molecule of neurotrophic signaling pathway was down‐regulated. However, the analysis of sequence comparison revealed that *2610307p16Rik* does not possess the capability to directly govern the expression of NTF3. Previous research has indicated a close association between FMR1 and the neurotrophic factor family, particularly BDNF and NTF3. The FMR1 protein has been observed to impede the transportation of *BDNF* mRNA and suppress its expression, thereby inhibiting synaptic function and electrical signal transmission.^[^ ^40^ ^]^ Nevertheless, the precise regulatory connection between FMR1 and NTF3 remains unresolved. It was observed that in *CASC15* over‐expressed SH‐SY5Y cells, FMR1 expression was significantly reduced and NTF3 expression was increased. In addition, the *CASC15* over‐expressed SH‐SY5Y cells combined with the over‐expression of FMR1 by lentivirus significantly reduced the expression level of NTF3. Put it all together, there is reason to believe that *CASC15* plays an important role in synaptic plasticity by regulating NTF3 expression, and FMR1 is a key bridge between these two components. Moreover, further studies demonstrated that the expression of FMR1 and NTF3 in hippocampus of *APP/PS1* AD mice treated with exosomes enriched with *2610307p16Rik* transcription were similar to the results in vitro. Therefore, exosomes rich in *2610307p16Rik* transcript can effectively improve the L&M ability of AD mice. Until now, the majority of evidence pertaining to the involvement of lncRNAs in neurogenesis, neuronal differentiation, and the regulation of neuronal development has primarily concentrated on their role within the nucleus.^[^ ^65^, ^66^ ^]^ In contrast to the aforementioned scenario where nuclear lncRNAs exert an influence on neuronal development and excitability, the functionality of cytoplasmic lncRNAs remains largely unexplored. However, it has been demonstrated that *cytoplasmic RNA1* (BC1) requiring coordination with FMR1 and factors influencing translation initiation is capable of modulating the translation process within neuronal dendrites, thereby directly impacting synaptic plasticity and memory.^[^ ^67^ ^]^ These findings imply that the role of lncRNAs is in line with its specific subcellular positioning. Nevertheless, this study did not investigate the exact subcellular localization of *CASC15* within the neurons of the CA1 region in the mouse hippocampus, nor did it delve into the precise mechanism by which *CASC15* effectively governs the FMR1‐NTF3 axis. The convergence of exosome biogenesis and the regulation of nerve cell secretory vesicles offers novel perspectives on the potential association between exosomes and the development of neurodegenerative disorders.^[^ ^68^, ^69^ ^]^ The successful vitro implementation of *2610307p16Rik* in the *APP/PS1* model demonstrated its efficacy; however, there remain significant scientific inquiries that need to be addressed in translational applications. For instance, our investigation examined the impact of elevated doses of *2610307p16Rik*, yet the potential involvement of physiological doses of *2610307p16Rik* and the improvement underlying the efficient targeting of *2610307p16Rik* exosomes to the hippocampus necessitate further investigation.

**FIGURE 8:**
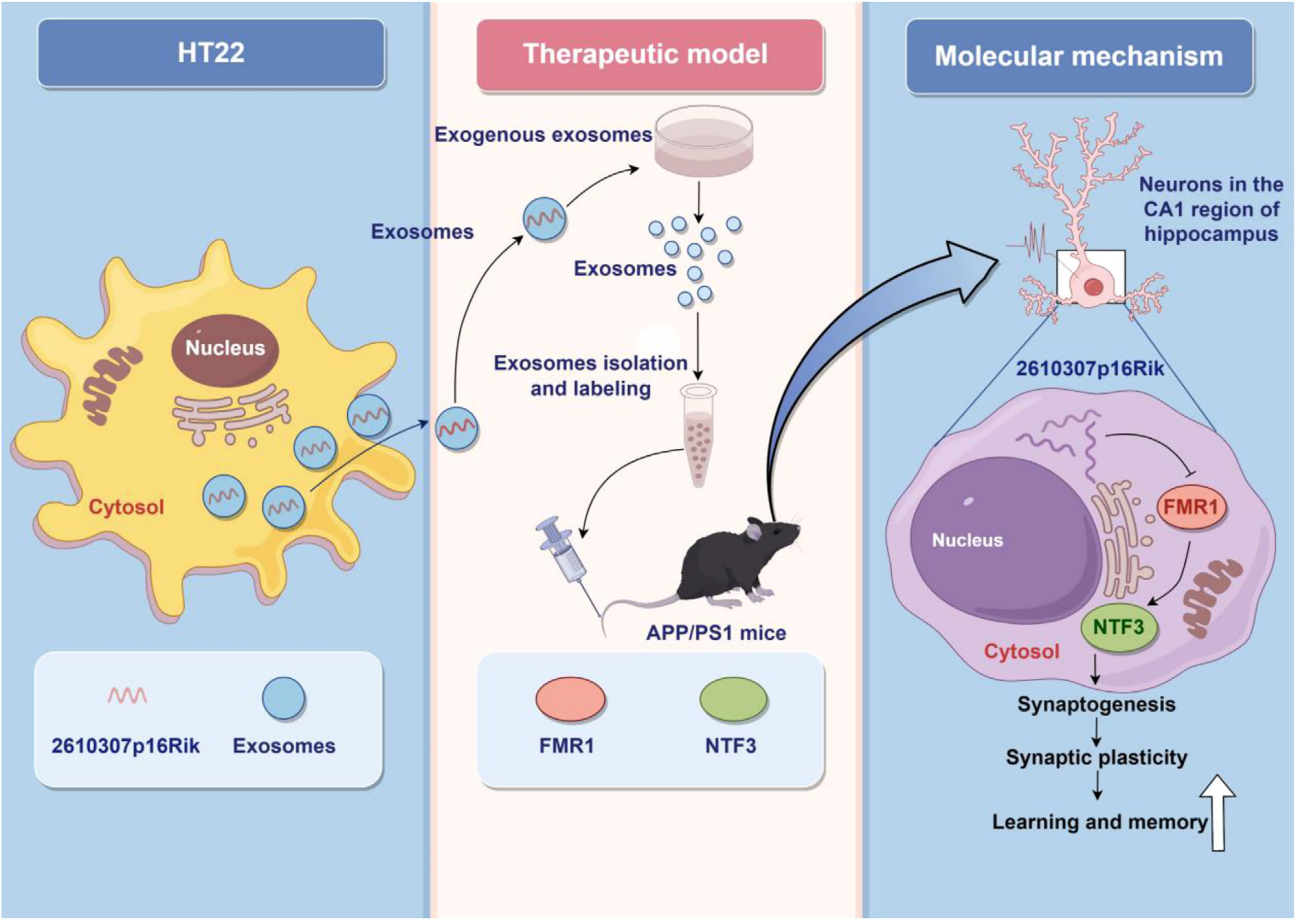
*CASC15* enhances learning and memory in mice by promoting synaptic plasticity in hippocampal neurons.

The overall findings suggest that a novel mechanism mediated by the lncRNA *CASC15* can regulates synaptic structure and function in hippocampal neurons (Figure 8). The elucidation of this new mechanism is valuable for understanding the function of *CASC15* in synaptic development and L&M processes, and is expected to be a new target for AD therapy.

## 4 EXPERIMENTAL SECTION

### 4.1 Methods

#### 4.1.1 Animal models

Approval for this study (No. IACUC‐20190950) was obtained from the Lab Animal Care and Use Committee at the Fourth Military Medical University. All experiments involving animals adhered to the guidelines outlined in the National Institutes of Health Guide for the Care and Use of Laboratory Animals. 8‐week‐old male C57BL/6 mice were sourced from the Fourth Military Medical University Animal Laboratory Center and were housed in a controlled environment with specific pathogen‐free conditions. The mice were maintained at a temperature range of 22–24°C with a humidity level of 50%, and were subjected to a 12‐h dark‐light cycle from 6 a.m. to 6 p.m. The 8‐week‐old *2610307p16Rik* and *APP/PS1* transgenic mice were procured from the Southern China Model Animal Center. Subsequently, the Animal Experimental Center of the Fourth Military Medical University undertook the responsibility of providing nourishment and facilitating breeding in accordance with the aforementioned feeding protocols. For the purpose of experimentation, both *2610307p16Rik* and *APP/PS1* transgenic mice, along with a cohort of wild‐type mice from the same batch, were employed as control subjects. During the in vivo exosome tail vein intervention therapy experiment, wild‐type mice were administered lipofectamine 2000 (Cat#11668030, ThermoFisher, USA) as a control group. The remaining three groups consisted of *APP/PS1* transgenic mice, with one group receiving lipofectamine 2000 (Cat#11668030, ThermoFisher, USA) intervention, another group receiving exosome intervention as the control group, and the final group receiving intervention with overexpressed exosomes of *2610307p16Rik*.

#### 4.1.2 In vitro cell‐lines

Mouse hippocampal neuronal HT22 cells and human neuroblastoma SH‐SY5Y cells were obtained from Cellbank of Chinese Academy of Sciences (Shanghai, China). Those cells were cultivated in DMEM (Cat#11885092, ThermoFisher, USA) supplemented with 10% fetal bovine serum (Cat#10091148, ThermoFisher, USA) and maintained in a standard humidified incubator with 5% CO_2_ at 37°C.

#### 4.1.3 Detection of gene expression by qRT‐PCR

TRIzol reagent (Cat#15596026, Invitrogen, USA) was used for total RNAs isolation from both mouse hippocampal neuronal HT22 cells and human SH‐SY5Y cells, as well as *2610307p16Rik* and *APP/PS1* transgenic mice hippocampus. cDNAs were synthesized by use of PrimeScript RT Master Mix (Cat#RR036A, TAKARA, Japan) in ProFlex PCR System (ABI, USA). qPCR was conducted via 7500 Fast Real Time PCR system (ABI, USA) with TB Green Fast qPCR Mix (Cat#RR430B, TAKARA, Japan). Quantification was performed using the 2^−ΔΔCt^ method, and all data were normalized to the level of the reference genes mRNA. All primer sequences are provided in File S1 in Supporting Information.

#### 4.1.4 Western blot

Cells were lysed with RIPA (Radio Immunoprecipitation Assay) lysis buffer (Cat#P0013C, Beyotime, China) on ice and cell debris was removed by centrifugation. Total protein extracts were quantified by Pierce BCA Protein Assay Kits (Cat#23225, ThermoFisher, USA). Electrophoreses were performed with different concentrations of BeyoGel Elite Precast PAGE Gel for Bis‐Tris System (Cat#P0867M, Beyotime, China), followed by electro‐transfer using hydrophobic polyvinylidene fluoride (PVDF) membrane (Cat#FFP22, Beyotime, China). Membranes were blocked in 5% non‐fat milk with phosphate buffered saline (PBS) Tween 20 (Cat#28352, ThermoFisher, USA), and the primary antibody (SNAP25 antibody, 1:400, Cat#3926, Cell Signaling Technology, USA); (Synaptotagmin‐1 Antibody, 1:400, Cat#3347, Cell Signaling Technology, USA); (PSD95 Antibody, 1:500, Cat#2507, Cell Signaling Technology, USA); (NR2B Antibody, 1:300, Cat#4207, Cell Signaling Technology, USA); (NR1 Antibody, 1:300, Cat# 5704, Cell Signaling Technology, USA); (GluA2+3 Antibody, 1:500, Cat#PA1‐4660, ThermoFisher, USA); (GluA2 Antibody, 1:500, Cat#13607, Cell Signaling Technology, USA); (GluA1 Antibody, 1:500, Cat#13185, Cell Signaling Technology, USA); (β‐Actin Antibody, 1:500, Cat#3700, Cell Signaling Technology, USA); (NTF3 Antibody, 1:300, Cat#PA1‐14861, ThermoFisher, USA); (FMRP Antibody, 1:500, Cat#4317, Cell Signaling Technology, USA) was added and co‐incubated at 4°C for 16 h. Corresponding secondary antibody (Goat anti‐Rabbit IgG (H+L) Secondary Antibody, HRP, 1:2000, Cat#31460, Cell Signaling Technology, USA); (Goat anti‐Mouse IgG (H+L) Secondary Antibody, HRP, 1:2000, Cat#31430, Cell Signaling Technology, USA) was introduced before bands visualization through Pierce ECL Western (Cat#32209, ThermoFisher, USA). Then, bands were quantified through Image J.

#### 4.1.5 RNA‐FISH

The brain of C57BL/6 mice were frozen and sectioned into slices of 15 µm thickness, and were hybridized with the denatured *2610307p16Rik* double‐stranded deoxyribonucleic acid (DNA) probes (Servicebio, China) by 70% formamide at 46°C for 1.5 h. The slides were then rinsed with diethypyrocarbonate (DEPC) water and put air‐dried. Finally, the slides were covered with anti‐fluorescence quencher (containing DAPI) and ready for photography.

#### 4.1.6 Morris water maze

The *2610307p16Rik* and *APP/PS1* transgenic mice were placed in the quadrant and trained once a day at each end of the 2 water entry points to allow the mice to find the platform. Escape time was set to 90 s. If the platform could not be found within 90 s, the latency was recorded for 90 s and the mouse was placed on the platform for 15 s to rest. After 5 days of training, the metrics of the hidden platform experiments, visual platform experiments, and space exploration experiments were recorded.^[^ ^70^ ^]^

#### 4.1.7 Novel object recognition task

Before training, *2610307p16Rik* and *APP/PS1* transgenic mice spent 30 min on an open‐field device. Mice were given 3 min to freely investigate two identical items, A and B, placed in fixed but different positions. After the warm‐up session, mice were returned to their original housing. In the last test, researchers swapped out one of the familiar objects A with a new one B, reintroduced the mice to the room, and monitored their behavior and how long it took them to explore the new addition. Murine short‐term memory was assessed using identification index and new objects exploratory number.

#### 4.1.8 Contextual fear conditioning and testing

Unconditioned stimuli (footshock) were administered to mice in an anechoic conditioning chamber (Med associates, USA) with a centrical platform surrounded by grid flooring with stainless steel strips. Before the experiment ever began, the mice were given time to adapt to their new surroundings. Mice who stayed on the floor throughout the series of pilot experiments got footshock (0.5 mA for 2 s), but mice that were placed on the platform were never exposed to the stimulus and so learned to avoid it. In the last experiment, a digital camera recorded how long each mouse spent on the platform. Murine contextual fear memory was assessed by use of freeze index and freezing time.

#### 4.1.9 Hematoxylin and eosin staining

Sagittal or coronal sections of mice, which had been embedded in paraffin, were utilized in this study. The sections were subjected to a sequential soaking and rinsing process involving anhydrous ethanol, 95% ethanol, and 75% ethanol solutions. Subsequently, the sections were stained with hematoxylin (Cat#C0105S, Beyotime, China) for a duration of 10 min. Following this, rinsing, differentiation, and fading were performed using a 1% hydrochloric ethanol solution. Subsequently, the sections were stained with 0.5% eosin (Cat#C0105S, Beyotime, China) ethanol for a duration of 3 min. Finally, the red coloration was removed by washing the sections in a 95% ethanol solution, and the sections were then treated with xylene to achieve transparency.

#### 4.1.10 Immunohistochemistry

Coronal sections of mice (15 µm in thickness), which had been embedded in paraffin, were utilized in this study. Coronal sections washed three times with PBS (pH 7.4) and permeabilized with 0.05% Triton X‐100 for 20 min in an ice bath. Samples were then blocked for 30 min at room temperature. After that, sections were incubated with primary antibodies (GFP antibody, 1:500, Cat#34664, Cell Signaling Technology, USA); (NTF3 Antibody, 1:200, Cat#MA5‐24883, ThermoFisher, USA); (NeuN antibody, 1:200, Cat#24307, Cell Signaling Technology, USA) at 4°C for 8 h. Subsequently, following the completion of sample slice cleaning, the second antibody (Goat anti‐Mouse IgG (H+L) Highly Cross‐Adsorbed Secondary Antibody, Alexa Fluor 488, 1:800, Cat#A‐11029, ThermoFisher, USA); (Goat anti‐Rabbit IgG (H+L) Cross‐Adsorbed Secondary Antibody, Alexa Fluor 568, 1:400, Cat#A‐11011, ThermoFisher, USA) should be incubated at ambient temperature while ensuring light avoidance for a duration of 3 h.

#### 4.1.11 Nissel staining

The coronal sections of mice (15 µm in thickness) were subjected to xylene for removal and cleaning, followed by immersion in 50%, 80%, and 95% ethanol solutions for dehydration. Subsequently, the sections were stained with a 1% toluidine blue dye at room temperature (25°C) for a duration of 90 min. After staining, the excess dye was gently rinsed off with clean water. Ultimately, the neuronal count was observed under a microscope and subjected to statistical analysis after sealing the sections with gum.

#### 4.1.12 Golgi staining

Mice brains were subjected to 100 µm vibration section, washed with deionized water, and then stained with FD Rapid GolgiStain Kit (Cat#PK401A, FD NeuroTechnologies, Columbia). After that, mixtures were washed and dehydrated by gradient. Finally, the slices were sealed for observation after xylene permeation.^[^ ^71^, ^72^ ^]^

#### 4.1.13 Electron microscopy

Mice brains were fixed with mixture containing glutaraldehyde. Sequence coronal sections of cerebral hippocampal tissue were collected, and the synaptic thickness and length of neurons were examined through a transmission electron microscope (JEM‐1230, JEOL, Japan).

#### 4.1.14 Miniature excitatory post synaptic current detection

Mice brains were subjected to section under ice bath condition, and the section thickness was 100 µm. The section was placed in PMSF of ice bath and treated with oxygen. mEPSCs were recorded at a holding potential of −70 mV in the presence of AP5 (50 µm, Cat#A5282, Sigma‐Aldrich, USA), picrotoxin (100 µm, Cat#S6100, Selleck, USA), and tetrodotoxin (TTX) (0.5 µm, Cat#554412, Sigma‐Aldrich, USA). The frequency and amplitude of mEPSCs were analyzed with Mini Analysis software.^[^ ^72^ ^]^

#### 4.1.15 RNA‐Seq

The hippocampus of *2610307p16Rik* transgenic mice was collected for total RNA extraction and RNA‐seq. RNA sequencing was done by Shanghai Applied Protein Technology. Genes with significantly different expression at *p* < 0.05 and log_2_fold change > 1 were identified between two *2610307p16Rik* transgenic mice groups.

#### 4.1.16 Virus construction and stereotaxic surgery

Two types of adeno‐associated virus (AAV), one for sh‐*FMR1* and the other one for negative control (NC), were both bought from OBIO (China), and stereotaxic surgery was carried out as previously described (Tan et al., 2016). SYN promoter‐dependent virus (0.5 µL, BrainVTA, China) was bilaterally injected into the hippocampus (anterior‐posterior [AP], −2.1 mm; medial‐lateral [ML], ± 1.8 mm; dorsal‐ventral [DV], −1.6 mm) at the speed of 0.1 µL min^−1^ and the needle was left in place for 10 min before being slowly withdrawn. After 3–4 weeks post‐surgery recover, *2610307p16Rik* transgenic mice were ready for usage of immunostaining.

#### 4.1.17 EV isolation and labeling

The supernatant of HT22 cell line overexpressing *2610307p16Rik* was collected and centrifuged at 300 × *g* for 10 min followed by 2000 × *g* for 10 min to remove cell debris. The supernatant was then transferred and filtrated through a 0.22 µm filter (Millipore, CA) followed by a round of centrifugation at 10,000 × *g* for 30 min. A third transfer was performed and the supernatant was centrifuged at 100 000 × *g* for 90 min. Pellets were collected and resuspended with 0.01 m PBS. ZETASIZER Nano series‐Nano‐ZS (Malvern, UK) and transmission electron microscopy (TEM) were used to determine the EVs size.^[^ ^73^, ^74^, ^75^ ^]^ DiI or DiR (Molecular Probes, MA) was incubated with EVs in PBS for mouse tail vein injection and labeling. The labeled mice were photographed in bioluminescent imaging by Xenogen In Vivo Imaging System (Caliper Life Science, MA).

#### 4.1.18 Statistical analysis

RNA‐Seq size factor adjust RPKM of postmortem brain tissue from AD patients were used for subsequent analysis of the sample data above. From the 219 AD samples, 50 AD samples were selected and then 50 controls with good correlation the AD samples are selected to detect gene expression using one‐tailed t‐test. TSpearman correlation analysis was used for analyzing the expression values of *CASC15*, FMR1 and NTF3. Data were displayed in a form of mean  ±  SEM. Student′s *t*‐test and two‐way ANOVA were performed to determine group difference. The validation of the results obtained from the two‐way ANOVA analysis was conducted using the Tukey test. Statistical analysis was performed using GraphPad and SPSS, and differences were considered significant if *p <  0.05, **p <  0.01, ***p <  0.001 or ****p <  0.0001.

## AUTHOR CONTRIBUTIONS

Yuankang Zou, Bo Gao, Jiaqiao Lu and Keying Zhang contributed equally to this work.

## CONFLICT OF INTEREST STATEMENT

Bo Gao is a member of the Exploration editorial board. The authors listed affirm that the publication of this article is not in contradiction with their interests.

## Supporting information

Supporting Information

## Data Availability

There was no disagreement between all authors regarding the final version of the manuscript. This article presents comprehensive information to substantiate its findings. The corresponding authors can provide the data upon reasonable request.
