# Supplementary material for: Long non‐coding RNA CASC15 enhances learning and memory in mice by promoting synaptic plasticity in hippocampal neurons
Source: Exploration (Beijing). 2024 Mar 28;4(6):20230154. doi: 10.1002/EXP.20230154 (PMC11655312; doi:10.1002/EXP.20230154)

**Supplementary1. 2610307p16Rik knockout did not affect brain development and limb motor ability in mice.** A. The expression of *2610307p16Rik* in mouse hippocampus was detected by ISH, scale bar: 20 µm. B. The expression of *2610307p16Rik* in the cortex and heart at different stages of nervous system development was measured by qRT-PCR (*U1snRNA* was used as control), and data were normalized to the expression of P0 Heart (mean ± SEM, n = 3). C. H&E staining was used to detect the sagittal section of the brain of *2610307p16Rik* wild-type and knock-out mice. D. H&E staining was used to detect the coronal section of the brain of *2610307p16Rik* wild-type and knock-out mice. E. Swimming speed in space exploration experiment of Morris water maze (mean ± SEM, n = 5). F. Total swimming distance in space exploration experiment of Morris water maze (mean ± SEM, n = 5).


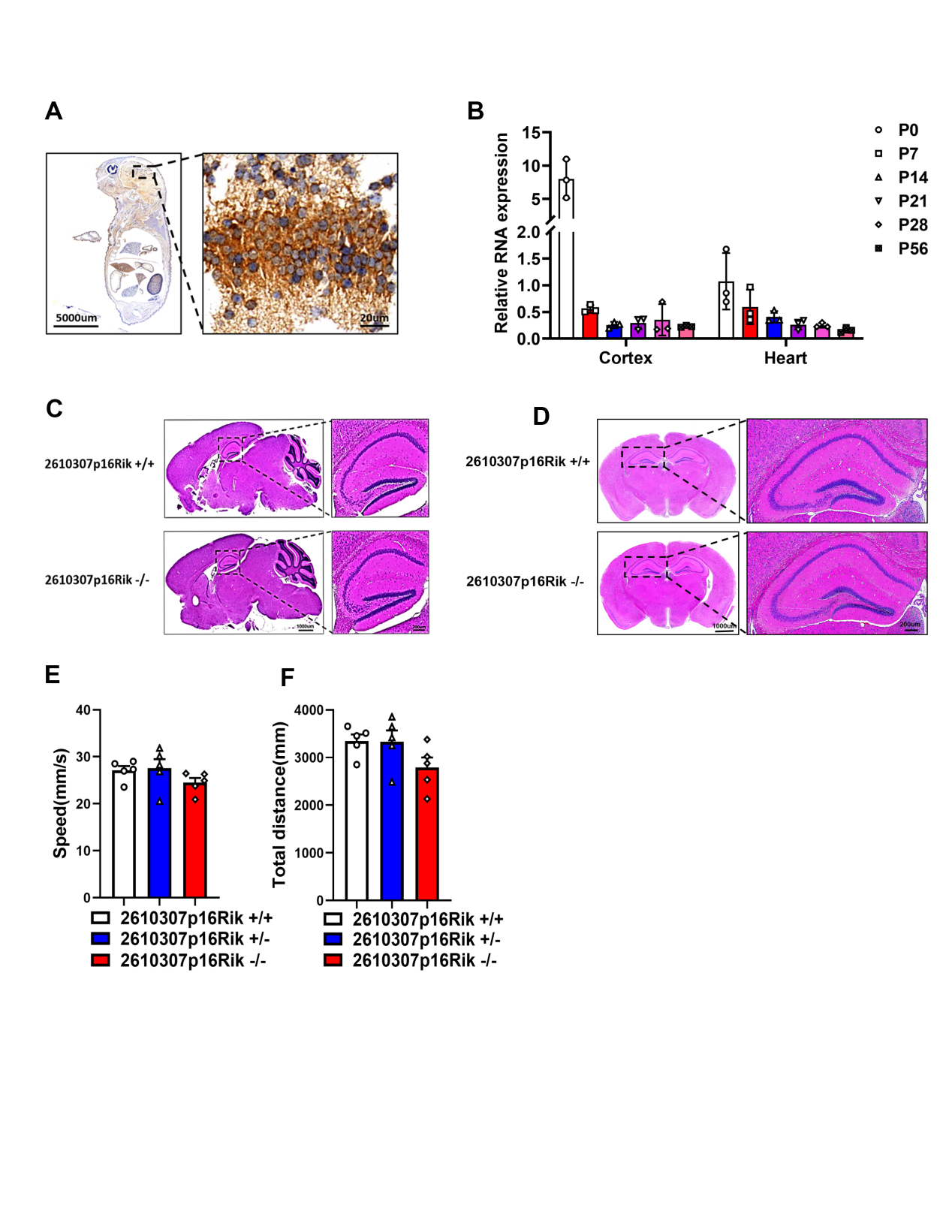


**Supplementary2. The expression of FMR1 and correlations among CASC15 and FMR1 in the clinical AD cases.** A. Relative RNA levels of *FMR1* in the 50 clinical AD cases and matched pairs. B. Pairwise correlations among *CASC15* and *FMR1* in the 50 clinical AD cases and matched pairs.


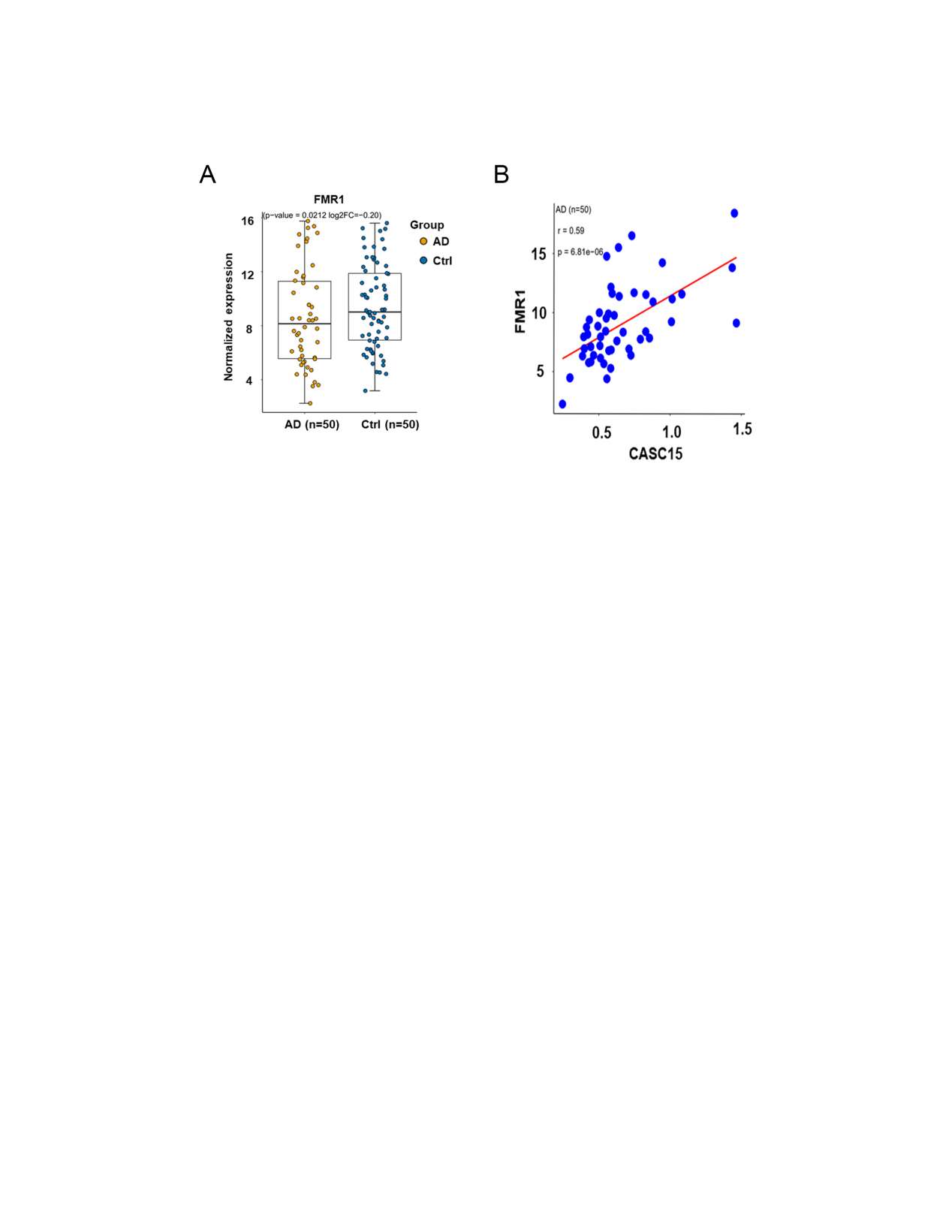


**Supplementary3. Effects of exosomes enriched with 2610307p16Rik on motor capacity and FMR1-NTF3 axis in mice.** A. qRT-PCR was used to detect the expression of *2610307p16Rik* in mouse hippocampal neuronal HT22 cells (*U1snRNA* was used as control), and data were normalized to the expression of Lv-Ctrl group (mean ± SEM, n = 3). Student′s t-test ***p* < 0.01. B. qRT-PCR was used to detect the expression of *2610307p16Rik* in exosomes retrieved from *2610307p16Rik* over-expression HT22 cell line (*U1snRNA* was used as control), and data were normalized to the expression of Lv-Ctrl group (mean ± SEM, n = 3). Student′s t-test *****p* < 0.0001. C. qRT-PCR was used to detect the expression of *2610307p16Rik* in hippocampal specimens from *APP/PS1* and control mice with different treatment methods (*U1snRNA* was used as control), and data were normalized to the expression of WT+Lipo group (mean ± SEM, n = 3). Two-way ANOVA ****p* < 0.001, *****p* < 0.0001. D. Co-staining for DiI with NeuN in the CA1 cortex showed the distribution of DiI-labeled exosomes at 48h after injection into the body through the tail vein, scale bar: 50 µm. E. Swimming speed of *APP/PS1* and control mice with different treatment methods in hidden platform trial of Morris water maze. F-G. qRT-PCR was used to detect the expression of *FMR1* and *NTF3* in the hippocampus specimens from *APP/PS1* and control mice with different treatment methods (*β-Actin* was used as control), and data were normalized to the expression of WT+Lipo group (mean ± SEM, n = 3). Two-way ANOVA **p* < 0.05, *****p* < 0.0001.


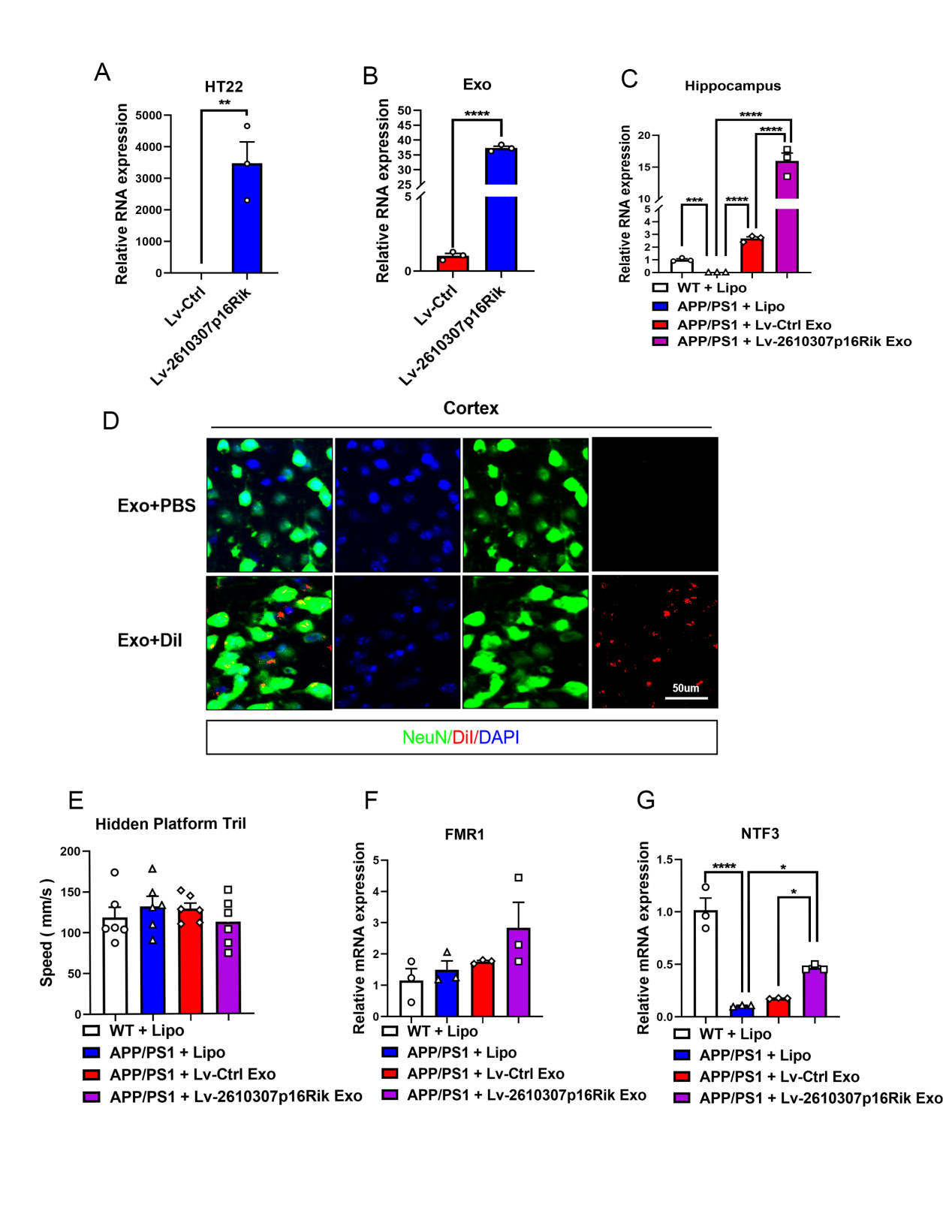

Supplement: Supplementary file 1 — Supporting Information [file EXP2-4-20230154-s001.docx]
